# Susceptibility to smartwatch based interventions in patients with MCI or dementia in a controlled environment

**DOI:** 10.1002/alz.089671

**Published:** 2025-01-09

**Authors:** Doreen Goerss, Stefanie Köhler, Stefan Teipel

**Affiliations:** ^1^ Department of Psychosomatic Medicine, Rostock University Medical Center, Rostock Germany; ^2^ Deutsches Zentrum für Neurodegenerative Erkrankungen e. V. (DZNE) Rostock/Greifswald, Rostock Germany

## Abstract

**Background:**

Promising elements of assistive technologies are available to help people with cognitive impairment in their daily lives. However, there has been limited research on how smartwatches can directly interact with persons who have cognitive impairments. We looked at the factors that affect the effectiveness of interventions provided via a smartwatch.

**Method:**

We designed two tasks delivered by smartwatch: A) drinking water and B) circling bells on a worksheet. We created two modes of intervention‐intensity affecting vibration, alarm sounds, text sizes and images. In case of failure, interventions were repeated up to three times. We observed n = 40 patients’ reactions to interventions via cameras and rated success on a 5‐step scale from 0 to 2. Patients were assigned (n = 20/20) either to mode “regular” or mode “intensive”. All patients received both tasks in the mode corresponding to the group. In both groups, half received the tasks in order AB, the other half in BA. Participants answered questionnaires and underwent neuropsychological testing. People were diagnosed with MCI (n = 12) or dementia (n = 28). We hypothesized that the mode, the sequence of tasks, age, affinity for technology (ATI) and cognitive status (MMSE) would influence the success of the interventions. We expected that intensive interventions would be more successful than regular interventions.

**Results:**

The groups statistically did not differ with respect to mean age, MMSE or ATI. The intensive group was more successful than regular group, for each task and global success score (mean 1.7, SD 0.3 vs. 1.2, SD 0.7., Welch t‐test p = 0.03). Testing of linear regression models showed that “mode” significantly increases model fit: H_1 age + sex + MMSE + ATI + task sequence + mode_ p = 0.045, adjusted R² = 19%, compared to H_0 age + sex + MMSE + ATI + task sequence_ p = 0.399, adjusted R² = 0.8%. Controlling for covariance (ANCOVA) showed only for “mode” a significant influence on success (p = 0.003) not of MMSE, sex or diagnosis (p<0.05, respectively).

**Conclusion:**

Tasks delivered from a smartwatch as intensive interventions are more likely to be solved by patients than regularly delivered ones. Intensity is more critical than the patient’s baseline status with respect to cognition and technology‐affinity.

